# Circulating Tumor Cell Enumeration for Serial Monitoring of Treatment Outcomes for Locally Advanced Esophageal Squamous Cell Carcinoma

**DOI:** 10.3390/cancers15030832

**Published:** 2023-01-29

**Authors:** Josephine Mun Yee Ko, Ka On Lam, Dora Lai Wan Kwong, Ian Yu-Hong Wong, Fion Siu-Yin Chan, Claudia Lai-Yin Wong, Kwan Kit Chan, Tsz Ting Law, Keith Wan Hang Chiu, Candy Chi Shan Lam, Jean Chrysei Wong, Henry Chun Hung Fong, Faith Sin Fai Choy, Andy Lo, Simon Law, Maria Li Lung

**Affiliations:** 1 Department of Clinical Oncology, School of Clinical Medicine, University of Hong Kong, Hong Kong, China; 2 Department of Surgery, School of Clinical Medicine, University of Hong Kong, Hong Kong, China; 3 Department of Diagnostic Radiology, School of Clinical Medicine, University of Hong Kong, Hong Kong, China

**Keywords:** circulating tumor cells (CTC), azygos vein blood, CTC clusters, longitudinal real-time monitoring, non-invasive biomarker, liquid biopsy, EMT, advanced ESCC, early prediction, prognosis

## Abstract

**Simple Summary:**

Esophageal cancer is an aggressive disease with dismal survival. Circulating tumor cells (CTCs) may provide useful information for the unmet needs of early predictive and prognostic biomarkers for therapeutic responses and disease progression. Our novel longitudinal CTC findings demonstrated the potential clinical utilities of real-time CTC monitoring for locally advanced esophageal squamous cell carcinoma (ESCC) patients along with curative resection treatment at multiple timepoints for prediction of treatment efficacies, prognostication, and real-time tracking of minimal residual disease for earlier detection of relapse. The presence of pre-surgery CTC clusters is independently associated with relapse. The unfavorable CTC status at any pre-treatment time and 1-/3-month post-surgery are independent prognosticators of disease progression and survival. For patients receiving chemoradiation therapy (CTRT), unfavorable CTC status at pre-/post-CTRT may serve as potential predictive biomarkers for monitoring treatment efficacy and guiding treatment decisions. Longitudinal CTC monitoring may be taken repetitively as non-invasive liquid biopsies to provide supplementary information with clinical imaging.

**Abstract:**

We aim to reveal the clinical significance and potential usefulness of dynamic monitoring of CTCs to track therapeutic responses and improve survival for advanced ESCC patients. Peripheral blood (PB) (*n* = 389) and azygos vein blood (AVB) (*n* = 13) samplings were recruited prospectively from 88 ESCC patients undergoing curative surgery from 2017 to 2022. Longitudinal CTC enumeration was performed with epithelial (EpCAM/pan-cytokeratins/MUC1) and mesenchymal (vimentin) markers at 12 serial timepoints at any of the pre-treatment, all of the post-treatments/pre-surgery, post-surgery follow-ups for 3-year, and relapse. Longitudinal real-time CTC analysis in PB and AVB suggests more CTCs are released early at pre-surgery and 3-month post-surgery into the circulation from the CTRT group compared to the up-front surgery group. High CTC levels at pre-treatments, 1-/3-month post-surgery, unfavorable changes of CTC levels between all post-treatment/pre-surgery and 1-month or 3-month post-surgery (Hazard Ratio (HR) = 6.662, *p* < 0.001), were independent prognosticators for curative treatment. The unfavorable pre-surgery CTC status was independent prognostic and predictive for neoadjuvant treatment efficacy (HR = 3.652, *p* = 0.035). The aggressive CTC clusters were more frequently observed in AVB compared to PB. Its role as an independent prognosticator with relapse was first reported in ESCC (HR = 2.539, *p* = 0.068). CTC clusters and longitudinal CTC monitoring provide useful prognostic information and potential predictive biomarkers to help guide clinicians in improving disease management.

## 1. Introduction

Worldwide, esophageal cancer (EC) is an aggressive common cancer and ranks seventh and sixth with an estimated 604,000 new cases and 544,000 deaths, respectively, in 2020 [1]. Esophageal squamous cell carcinoma (ESCC) is the predominant histological subtype in certain regions of Asia, Africa, and Europe, whereas esophageal adenocarcinoma (EAC) occurs more frequently in North America and parts of Europe [1]. In Asian countries, including China, ESCC accounts for >90% of EC. Most ESCC patients are asymptomatic and diagnosed at an advanced stage leading to dismal survival. Early tumor recurrence and distant metastasis after surgery make ESCC a major cause of cancer deaths [2,3,4].

In the metastatic cascade transforming localized tumors into systemic disease, tumor cells shed from primary sites and disseminate into the circulating blood [5,6]. Circulating tumor cells (CTCs) or cancer cells undergo partial epithelial-mesenchymal-transition (EMT) to gain motility and invasiveness [7]. Early detection and longitudinal monitoring of CTCs during disease progression provide an opportunity to prevent overt metastasis and improve patient survival. Despite technological advancements in blood CTC isolation, their rarity and heterogeneity make their isolation challenging [5,6]. Earlier ESCC studies detected CTCs using molecular assays to examine the mRNA level by RT-PCR with positive detection rates ranging from 25 to 60% [8,9,10,11,12,13,14]. CTCs could be better predictors of relapse compared to serum tumor antigens such as squamous cell carcinoma antigen (SCCA) and carcinoembryonic antigen (CEA) [10,12]. The CellSearch system is the only FDA-approved EpCAM-based CTC isolation platform, but it is unable to capture the aggressive subpopulation of CTCs undergoing EMT or those expressing a low level of EpCAM. Previous ESCC studies using the CellSearch positive selection approach reported CTC detection rates ranging from 18% for resectable cases to 27.8–50% for unresectable cases treated with chemotherapy (CT) or chemoradiation therapy (CTRT) cases [15,16,17,18,19]. The recent detection of ESCC CTCs showed improved sensitivity utilizing negative selection strategies, such as depletion of white blood cells with CD45-conjugated magnetic bead and marker-independent approaches such as isolation by size of epithelial tumor cells (ISET) or fluid-assisted separation technique (FAST) [16,17,20,21,22,23]. Our previous CTC work in multiple cancers, including advanced ESCC, has successfully demonstrated CTC enrichment by size separation utilizing centrifugal microfluidic devices [24,25,26,27,28,29]. Although numerous earlier ESCC studies demonstrated its predictive and prognostic values at a single timepoint CTC level at baseline or post-treatment, comprehensive longitudinal CTC monitoring with multiple timepoints along the treatment course has yet to be evaluated for its clinical utility in terms of real-time monitoring for disease management [8,9,10,11,15,17,18,19,20,23,30,31,32,33]. One of the main goals was to reveal the clinical significance and the potential usefulness of dynamic monitoring of CTCs to track therapeutic responses for advanced ESCC to improve patient survival. Another aim of the current study was to provide proof-of-concept evidence that high numbers of CTCs are released into the blood circulation from the tumor and to determine whether more CTCs were released from the group of patients treated by the neo-adjuvant CTRT compared to the upfront surgery group.

Earlier ESCC CTC studies demonstrated the prognostic role of CTCs focusing on single CTCs [8,9,10,11,15,17,18,19,20,23,30,31,32,33]. Recent technological advancement demonstrated the additional prognostic value of CTC clusters, especially in metastatic breast cancer, with up to a 100-fold higher metastatic potential compared to individual CTCs [34,35,36]. However, the prognostic role of CTC clusters in ESCC remains unknown. We aim to investigate whether the presence of CTC clusters or high CTC levels provides critical information for the stratification of treatment decisions. The curative treatment options of locally advanced ESCC patients include upfront surgery, neoadjuvant CTRT treatment prior to surgery, or radical CTRT. For instance, the detection of CTCs in the peripheral blood before upfront surgery may indicate the need for neo-adjuvant treatment prior to surgery. The presence of CTCs after surgery or pre-surgery CTC clusters may indicate the need for adjuvant treatment. Serial CTC analysis for patients receiving neoadjuvant and surgery treatment may impact the timing of surgery and post-surgery adjuvant regimens. The current study suggested the clinical utility of CTC longitudinal monitoring as a non-invasive early predictive biomarker of treatment outcome or independent prognosticator of disease progression and survival of locally advanced ESCC patients undergoing curative treatments. The current comprehensive findings indicate the usefulness of several timepoints taken at all pre-treatment, post-treatment/pre-surgery, 1-month, and 3-month post-surgery during longitudinal real-time CTC monitoring.

## 2. Materials and Methods

### 2.1. Patients and Sample Collection

The current prospective study recruited 88 newly diagnosed histologically confirmed locally advanced ESCC stages I-III patients at Queen Mary Hospital between 2017 and 2022 (Figure S1). The enrolled patients (cT1/N+ or cT2-4a/N0-3/M0) were treated by curative upfront surgery with/without adjuvant CTRT (*n* = 33), first-line Chemoradiotherapy for Oesophageal Cancer followed by Surgery Study (CROSS) regimen (*n* = 48), or radical CTRT only (*n* = 7). CT treatment included paclitaxel and platinum-based anti-cancer drugs. A total of 389 peripheral blood samples were collected from 88 patients (Supplementary Table S1). One patient was recruited twice for two lines of treatment. The timeline for serial peripheral blood specimen sampling taken for CTC enumeration at baseline before treatment (CTC1), after two cycles of CT (pre-III) (CTC2), and at the end of neoadjuvant CRT/pre-surgery (CTC3), and post-surgery (CTC4–13) is illustrated in Supplementary Figure S2. For the first-year post-surgery follow-up, blood samples were taken at one-month post-surgery (CTC4) and then at 3/6/9/12 months post-surgery (CTC5-8), then half yearly from 1.5 to 3 years (CTC9–12) and at relapse (CTC13) to correlate with the positron emission tomography-computed tomography (PET/CT) imaging to evaluate clinical response. Guidelines from the European Organisation for Research and Treatment of Cancer (EORTC) were followed to define tumor response by PET-CT imaging [25]. Complete metabolic response (CMR) is defined as complete resolution of FDG uptake in all lesions, partial metabolic response (PMR) has ≥25% reduction in the sum of SUVmax after more than one cycle of treatment, and progressive metabolic disease (PMD) is defined as having ≥25% increase in the sum of SUVmax or appearance of new FDG-avid lesions. Stable metabolic disease (SMD) is defined as being neither CMR, PMR, nor PMD. Progression of disease (PD) included PMD, while non-PD included CMR, PMR, and SMD. Informed consent for sample collection from the ESCC patients was obtained according to protocols approved by the Institutional Review Board (IRB) of the University of Hong Kong/Hospital Authority Hong Kong West Cluster (HKU/HA HKW IRB) (ethic code: UW17–187). The study was performed in accordance with the Declaration of Helsinki. Both peripheral blood (PB) and azygos vein blood (AVB) samples for 13 patients were collected prior to surgery to compare CTC counts. Two patients were excluded from correlation analysis with survival due to the presence of dual primary tumors or liver cancer relapse (Figure S1).

### 2.2. CTC Enrichment and Enumeration

PB or AVB samples (7.5 mL) were collected in STRECK tubes kept at 4 °C and processed for CTC enumeration within 72 h. After red blood cell lysis, larger CTCs were enriched and separated by size from smaller white blood cells by centrifugal force with CTChip^®^FR1 microfluidic chips (ClearCell^®^FX1 System, Biolidics, Singapore) and immunofluorescence (IF) enumeration, as previously described [24,25,26,27,29]. Epithelial CTCs (CTC_E_) were identified by standard IF procedures with pan-CK/EpCAM/MUC1-Alexa 488 conjugated (Pan-Keratin C11, Cell Signaling, Beverly, MA, USA; Pan-Cytokeratin AE1/AE3, eBioscience, San Diego, CA, USA; EpCAM VU1D9, Cell Signaling, Beverly, MA, USA; CD227/Mucin1 SM3, eBioscience, San Diego, CA, USA) and CD45-APC conjugated (BD Pharmingen, San Diego, CA, USA) antibodies, as previously described [24,25,27]. The mesenchymal CTCs (CTC_M_) were identified with vimentin-DyLight 550 conjugated antibodies (2A52, Novus, San Diego, CA, USA). All antibodies were diluted to 1:100. The slides were scanned to obtain cell images by Cytation 5 Cell Imaging Multi-Mode Reader (BioTek, Centennial, CO, USA, San Diego, CA, USA). The potential CTC images were analyzed by imaging software 1-Click Plus (OncoSeek, V2022.10, www.oncoseek-hk.com, accessed on 31 October 2022) and confirmed by manual inspection. Cells staining DAPI^+^/CD45-/(pan-CK/EpCAM/MUC1)^+^ are considered CTC_E_ and DAPI^+^/CD45-/vimentin^+^ cells are considered CTC_M_. PB samples from ten healthy individuals were included as references for both CTC_E_ and CTC_M_ counts.

### 2.3. Cell Spiking Experiments for Reproducibility of Recovery Rate

We routinely monitored the CTC recovery rate with spike-in experiments using two ESCC cell lines of different sizes (KYSE30 and KYSE270) into 7.5 mL healthy blood, as previously described [24,25]. The mean recovery rate of mean input of 197 cells of each cell line was 73.43% ± 8% and 62% ± 10% in 10 experiments for the larger KYSE30 cells (average size ~17 µm) and KYSE270 (average size of ~15 µm), respectively, to ensure reproducibility of CTC enrichment.

### 2.4. Statistical Analysis

Pearson chi-square and Fisher’s exact tests were used for comparison between CTC status and categorical clinicopathological factors. Student’s t test was used for comparison of mesenchymal and total CTC numbers. The CTC status was classified into an unfavorable group (≥3 CTC_E_ or CTC_E+M_) or favorable group (0–2 CTC_E_ or CTC_E+M_) for comparison of progression-free survival (PFS) and overall survival (OS) with threshold determined by receiver operating characteristic (ROC) curve analysis considering sensitivity and specificity (Table S2). PFS or OS were defined by the period between surgery date or CTRT start to date of clinical progression or date of death, respectively. Survival analysis of baseline, pre- and post-surgery CTC status was compared by Kaplan–Meier (KM) curves and log-rank tests. KM analysis was used to estimate disease progression of PFS and OS according to CTC status. The KM survival curves were compared using log-rank tests. The clinical parameters, baseline and pre- and post-surgery CTC status, and the CTC cluster status were subjected to univariate COX analysis for both PFS and OS. Multivariate COX analysis was performed for the significant parameters in the univariate analysis. Survival analyses were performed with SPSS v26 (SPSS Inc, IBM Corporation, Armonk, NY, USA) or the survival package in R [37,38]. The Schoenfeld’s global test ensured no violation of proportional hazards assumption. Cox regression models with gender as time-varying covariate were used to calculate hazard ratios (HR) of PFS and OS [39]. Two-sided *p* values < 0.05 were considered statistically significant. Scatter plots and histograms were generated using GraphPrism Version 6.01 for Windows, (GraphPad Software, San Diego, CA, USA, www.graphpad.com, accessed on 31 October 2022).

## 3. Results and Discussion

### 3.1. Patients Characteristics

Patient selection and the timeline for serial real-time CTC monitoring along the treatment course are detailed in Supplementary Figures S1 and S2, respectively. Detailed clinicopathological characteristics of 88 locally advanced ESCC patients receiving curative treatment are summarized in Table 1. The majority of patients were male (64/86, 74.4%). The patients had a median age of 69 ± 9.7, ranging between 30 and 87; 48 (55.8%) patients had disease progression; 35 (40.7%) patients died. The median PFS and OS were 374 ± 375.4 days and 520.5 ± 355.4 days, respectively. PFS and OS ranged between 19 and 1527 days. The patients receiving curative resection after neoadjuvant CTRT vs. upfront surgery did not differ in PFS and OS (Figure S3). Therefore, they were combined for prognostication analysis in Section 3.5 and Section 3.6.

### 3.2. Resectable Patients Treated by Pre-Surgery Treatment Had Higher CTC Counts

#### 3.2.1. Patients Treated by Neoadjuvant CTRT Associated with High CTC Level at 3-Month Post-Surgery

Locally advanced ESCC patients with large tumors are usually treated by neoadjuvant CTRT to downstage the tumors before surgery. Our serial CTC analysis findings suggested patients receiving neo-adjuvant CTRT treatment associated with significantly higher total CTC5_E+M_ at 3M post-surgery (CTRT group: 25.7%, 9/35 vs. Upfront group: 0%, 0/23, *p* = 0.008) and a trend of higher total CTC4_E+M_ at 1M post-surgery (CTRT group: 29.7%, 11/37 vs. Upfront group: 11.5%, 3/26, *p* = 0.126, Table 1) compared to those treated by upfront surgery. At the end of CTRT treatment before surgery, the neo-adjuvant CTRT group also had a trend of high epithelial CTC3_E_ associated with advanced pathological T (pT) (advanced pT 25%, 4/16, vs. early pT, 5.3%, 1/19, *p* = 0.156) and pN after resection (late pN 31.3%, 5/16, vs. early pN, 5%, 1/20, *p* = 0.069) (Table 1). There was no significant association between epithelial CTC_E_ and total CTC_E+M_ levels at baseline, 1M, and 3M post-surgery with age, sex, differentiation status, primary tumor location, stage at diagnosis, and distant metastasis status (Table 1). The CTC longitudinal analysis suggested advanced ESCC patients receiving pre-surgery CTRT treatment released more CTCs early in the first few months post-surgery compared to upfront surgical treatment.

#### 3.2.2. Proof-of-Concept Experiment: More CTCs Released into Azygos Vein Blood Versus Peripheral Blood in Patients Treated by Neoadjuvant CTRT Followed by Curative Surgery

**Figure 1:**
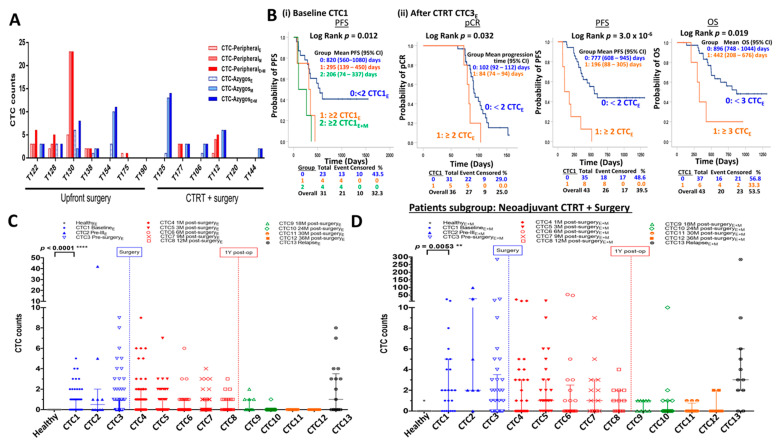
Summary of CTC counts of resectable ESCC. (**A**) Enumeration of the CTC_E_ and CTC_E+M_ from the azygos vein and peripheral bloods on the day before surgery from 13 ESCC patients. (**B**) Kaplan–Meier survival analysis of patients treated by CTRT + surgery of (**i**) CTC1 enumeration at baseline with PFS, and (**ii**) CTC3_E_ enumeration at the end of CTRT with pathological complete response (pCR), PFS, and OS. Summary of the enumeration of (**C**) of epithelial CTC_E_ (EpCAM/pan-CK/MUC1) and (**D**) total CTC_E+M_ with additional vimentin marker for CTC1–CTC13 in ESCC patients treated by curative treatment and normal individuals. The median with interquartile range (IQR) is indicated by error bar. Details of CTC1-CTC13 refer to Supplementary Figure S2. ** *p*-value < 0.01, **** *p*-value < 0.0001.

Compared to peripheral circulation, CTCs from colorectal cancer patients are more frequently released into the hepatic portal vein [40]. As a proof-of-concept experiment for higher CTC release frequency from ESCC patients treated by neoadjuvant CTRT treatment into the circulation, we performed a pilot study to compare CTC enumeration between the AVB and PB in the following two groups of patients (*n* =13) collected on the same day before surgery: upfront surgery (*n* = 7) as the reference group and neoadjuvant CTRT followed by surgery (*n* = 6) as the test group. AVB was chosen because it is located medial to the esophagus for the transportation of deoxygenated blood into the superior vena cava vein. CTCs were detected in either AVB or PB in 84.6% (11/13) of patients. A higher frequency of positive CTC detection rate of 76.9% (10/13) was observed in AVB compared to 53.8% (7/13) in PB (Figure 1A and Figure S5). Interestingly, higher CTC level in the AVB compared with PB was observed in 80% (4/5) patients with pre-surgery CTRT treatment compared to only 16.7% (1/6) patients in the upfront surgery group (Fisher’s exact test, *p* = 0.08, two-tailed) (Figure 1A). We observed a trend of higher mesenchymal CTCM (*t* test, *p* = 0.15) in the AVB vs. PB obtained on the same day for patients undergoing surgery after neoadjuvant treatment but not for patients receiving upfront surgery. This result, although based on a small sample cohort, provides novel insight suggesting the hypothesis that more aggressive mesenchymal CTCs are released into AVB for patients receiving neoadjuvant treatment. The AVB findings, taken together with the earlier PB blood findings in Section 3.2.1, lead to the speculation that more EMT-positive CTCs may be released early at the end of CTRT/pre-surgery, at 1-month and 3-month post-surgery in patients upon neoadjuvant CTRT therapy and migration of these CTCs to distant organs may occur. This might potentially facilitate metastatic seed dormancy and transformation into cancer stem cells at the relapse sites during the latency period. More validation studies and independent centers with larger sample cohorts are warranted to confirm our hypothesis generated from the current pilot study and preliminary findings.

#### 3.2.3. Early Predictive and Prognostic Biomarkers for Pre-Surgery CTRT Treatment Efficacy

There is an unmet need for predictive and prognostic markers to assess pre-surgery treatment efficacy. For the group of patients receiving pre-surgery treatment with evaluable baseline CTC1 counts (*n* = 31), those patients in the high-risk groups 1 (high level of epithelial CTC_E_) and 2 (high level of total CTC_E+M_) at baseline had a significantly shorter PFS (*p* = 0.012) than those patients in the low-risk group 0 (low level of epithelial CTC_E_) (Figure 1B(i)). This is the first report of the prognostic role of baseline CTCs isolated by FAST based on the CTC size for resectable cases treated with CTRT and concordant to previous reports that used positive or negative immunomagnetic isolation followed by flow cytometry [17,18]. Among them, a subset of nine patients also receiving adjuvant CTRT, those patients with positive CTC2_E_ collected at pre-III after two cycles of chemotherapy, significantly correlated with poor treatment response defined by the stable disease (SD) and PD results of the end of CTRT imaging reassessment (Supplementary Table S3, *p* = 0.048). This current pilot study first suggests the hypothesis that epithelial CTC2_E_ at pre-III may serve as a potential early predictive biomarker for pre-surgery CTRT treatment efficacy. Our findings also are the first to report CTCs detected by the FAST strategy for the 36 patients treated by neoadjuvant CTRT and surgery subgroup, high epithelial CTC3_E_ level at the end of neoadjuvant CTRT significantly associated with non-pathological complete response (non-pCR) (*p* = 0.032), shorter PFS (*p* = 3.0 × 10^−6^), and OS (*p* = 0.019) (Figure 1B(ii)). Our current findings show that the CTC status of both baseline and end of CTRT are useful for the evaluation of the efficacy of neoadjuvant CTRT and surgery and provides useful predictive and prognostic information. The predictive role of post-treatment CTC for the efficacy of neoadjuvant CTRT is in line with an earlier study that isolated mesenchymal CTCs by filtration with the CanPatrol system [41]. Previous ESCC CTC studies also demonstrated the prediction of treatment response and prognostic biomarker role of change of CTC status before and after treatment [8,15,19]. Due to the missing baseline CTC data in 20 patients in this subgroup, meaningful analysis of the change of CTC status before and after treatment was precluded. Our preliminary observations were limited by the small sample size, especially the predictive role of CTC2_E_ for patients with both neo-adjuvant and adjuvant treatments, and warrant validation studies with larger sample cohorts to guide therapeutic options to clinical care.

### 3.3. Longitudinal CTC Enumeration Analysis from Baseline to 3-Year Post-Surgery along Course of Curative Treatment

#### 3.3.1. Dynamics of Epithelial CTC Counts at Baseline, during and Post-Treatment

Compared to healthy individuals, ESCC patients had a significantly higher positive detection rate at baseline epithelial CTC1_E_ (24/61, 39.3% vs. 0/10, 0%, Fisher’s exact test, *p* < 0.0001, Figure 1C). Positive CTC_E_ counts ranged from 39.3% (24/61) at baseline CTC1_E_, reached a peak level of 50% (5/10) at pre-III CTC2_E_, but went down to 33.3% (15/45) at pre-surgery CTC3_E_, 31–27.7% at CTC5_E_–CTC6_E_ during post-surgery 3M to 6M and was highest (52.9%, 9/17) at relapse. The positive CTC detection frequency of post-surgery epithelial CTC4_E_, CTC7_E_–CTC9_E_ at 1M, 9M to 18M post-surgery, were slightly higher or similar to baseline, and then went down to 0–5.9% at CTC10–CTC12 during 24M to 36M post-surgery (Figure 1C and Supplementary Table S4). At 1 month after resection, the unexpectedly higher positive detection rate at epithelial CTC4_E_ compared to baseline should be interpreted with caution, as epithelial cells may be released into circulation from the traumatized tissues.

#### 3.3.2. Comparing Longitudinal Dynamics of Epithelial and Total CTC Counts

**Figure 2:**
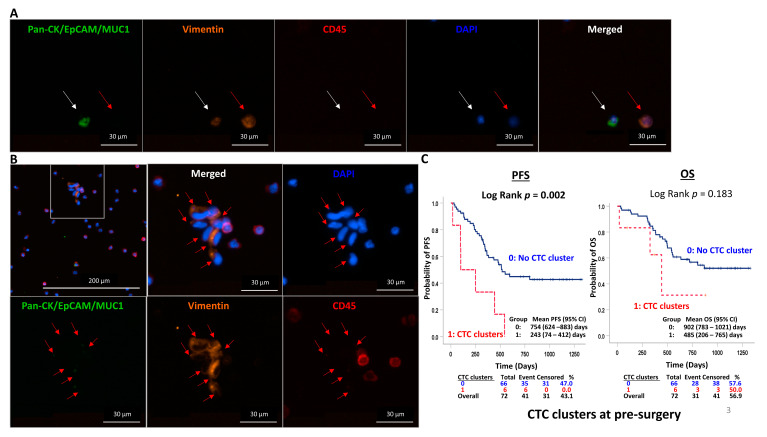
Double positive CTC, CTC_M_ and CTC cluster. (**A**) Representative double-positive epithelial and mesenchymal CTCs (white arrow) and (**B**) mesenchymal CTCs (red arrows) in cluster enriched from the pre-surgery blood of ESCC patient T130. (**C**) Kaplan–Meier curve analysis indicated a shorter PFS and a trend of shorter OS between patients with versus without pre-surgery CTC clusters. PFS: progression-free survival, OS: overall survival.

Mesenchymal-like CTC subpopulations may down-regulate epithelial markers, such as EpCAM and keratin, and up-regulate mesenchymal markers, such as vimentin [42]. Cancer cells undergo EMT and become highly invasive with greater metastatic potential. Hence, enumeration of total CTC_E+M_ with both epithelial markers and vimentin was performed in a subset of 66 patients (204 PBs) to capture more aggressive CTC subpopulations. Compared to healthy individuals, ESCC patients had a significantly higher positive detection rate of total CTC1_E+M_ at baseline (16/23, 69.6% vs. 1/10, 10%, Fisher’s exact test *p* = 0.0053, Figure 1D). Figure 2A shows representative images of mesenchymal CTC_M_ (Pan-CK/EpCAM/MUC1)-/Vimintin+/CD45-/DAPI+) and double-positive CTC (Pan-CK/EpCAM/MUC1)+/Vimintin+/CD45-/DAPI+). Positive CTC_E+M_ counts, including epithelial, mesenchymal, or double positive CTCs, ranged from 69.6% (16/23) at CTC1_E+M_, reached a peak level of 85.7% (6/7) at pre-III CTC2_E+M_, but decreased to 63.6% (14/22) at CTC3_E+M_, further dropped to 46.9% at CTC4_E+M_ and remained high 72.4% (19/27) at CTC5_E+M_ and 81.8% (9/11) at relapse (Figure 1D and Supplementary Table S5). The range, median, and positive detection rates of CTC_E_ and CTC_E+M_ at CTC1-CTC13 are summarized in Supplementary Tables S4 and S5, respectively. In general, positive detection rates and mean CTC_E+M_ were comparatively higher at all CTC timepoints, demonstrating a similar pattern of dynamic change compared to CTC_E_ rates. At baseline and at 3M post-surgery, significantly higher total CTC1_E+M_ (*p* = 0.021) and CTC5_E+M_ (*p* = 0.01) were observed, while higher trends, but no significant difference of total CTC4_E+M_ (*p* = 0.12), CTC6_E+M_ (*p* = 0.14), and CTC7_E+M_ (*p* = 0.13), at 1M, 9M, and 12M post-surgery, were observed (Figure S4). The CTC detection rate of 70% total CTC_E+M_ at baseline by FAST was higher, while a similar frequency of 39% of baseline epithelial CTCE, compared to that reported by the CellSearch platform, was observed [15,16,17,18,19]. The current study provides the first comprehensive longitudinal monitoring of the dynamics of CTCs for ESCC patients undergoing curative surgical treatment at regular intervals up to three years post-surgery. Our findings provide useful information for the evaluation of the critical timepoints for longitudinal CTC monitoring. The addition of a vimentin marker to track mesenchymal CTCs may improve the sensitivity for CTC detection. Our data agree with previous studies suggesting TWIST-positive CTCs undergoing EMT from ESCC patients are common [22,41].

### 3.4. Pre-Surgery CTC Clusters Associated with Earier Disease Relapse

Despite CTC clusters in circulation being a rare event, their role in predicting poor prognosis was demonstrated in various cancers [43,44,45,46]. Only one earlier ESCC study isolated CTCs by the ISET method reported 4.9% (3/61) circulating tumor microemboli (CTM) [16]. CTC clusters were defined as clusters of 2–50 cancer cells, as reported in earlier studies using different CTC isolation platforms in patients with metastatic epithelial cancers [47,48,49]. Our study observed about 2-fold higher frequency of 8.3% (6/72) pre-surgery CTC clusters and first demonstrated its prognostic role in ESCC patients receiving curative surgery. A representative CTC cluster is shown in Figure 2B versus a single CTC_E+M_ and CTC_M_ in Figure 2A. Among the 389 PB samples collected for CTC enumeration, CTC clusters occurred significantly more frequently in the AVB (2/13, 15.4%) compared to the PB (10/389, 2.57%, *p* = 0.05). However, these findings are limited by the significantly different sample sizes between the PB and AVB due to the limited availability of AVBs for this study. Future validation studies with a larger sample size of AVB are warranted. Patient T113, a 64-year-old male treated by CTRT and surgery, developed an early relapse after 101 days, and he died after 326 days. This was an example to illustrate the clinical usefulness of longitudinal monitoring of CTC clusters during his course of disease. Despite the fact that CTC cluster occurs rarely, recurrent CTC clusters were detected in this patient in both PBs taken at baseline and end of CTRT at pre-surgery timepoints. Recurrent CTC clusters at CTC1, CTC2, and CTC3 were also evidenced in three more patients treated by neoadjuvant CRT and surgery (T112, T125, and T193, Supplementary Table S6). Longitudinal monitoring of another patient, T108 (male/47), collected eight PB samples over a 15-month period. He received three lines of treatment (neoadjuvant CRT, palliative CRT, and immunotherapy) and developed recurrence with regional, liver, bone, and lung metastases. CTC cluster was identified in the final PB sample prior to death. Importantly, the six patients with pre-surgery CTC clusters all developed relapse and had statistically significant shorter PFS (*p* = 0.002) and a trend of shorter, but not significantly different OS (*p* = 0.183), compared to those without CTC clusters (Figure 2C). In the CTC3CL COX regression model, the presence of pre-surgery CTC cluster (HR = 2.539, *p* = 0.068) and pT were independent prognostic indicators of PFS (Table 2). However, the presence of CTC clusters did not correlate with OS in KM survival and the CTC3CL COX analysis, possibly due to insufficient length of follow-up. Our preliminary findings suggest longitudinal monitoring of CTC clusters at baseline, pre-III, and at the end of CRT pre-surgery for this group of patients may be beneficial for prognostication. Independent validation studies are required to substantiate our observations.

### 3.5. Serial CTC Enumeration at Multiple Timepoints Is Associated with Adverse Outcome

**Figure 3:**
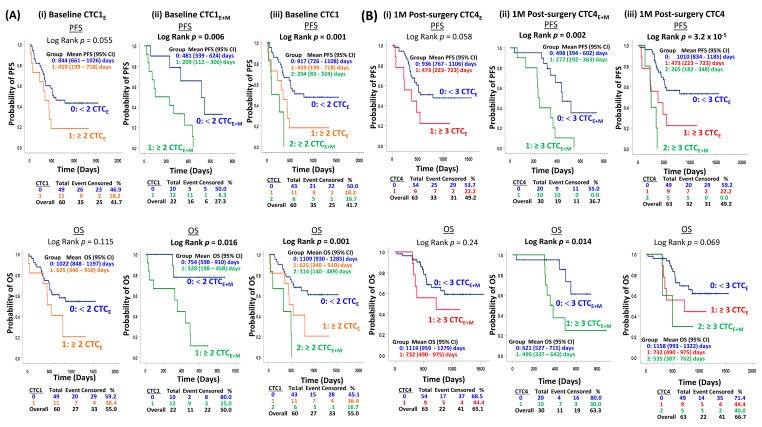

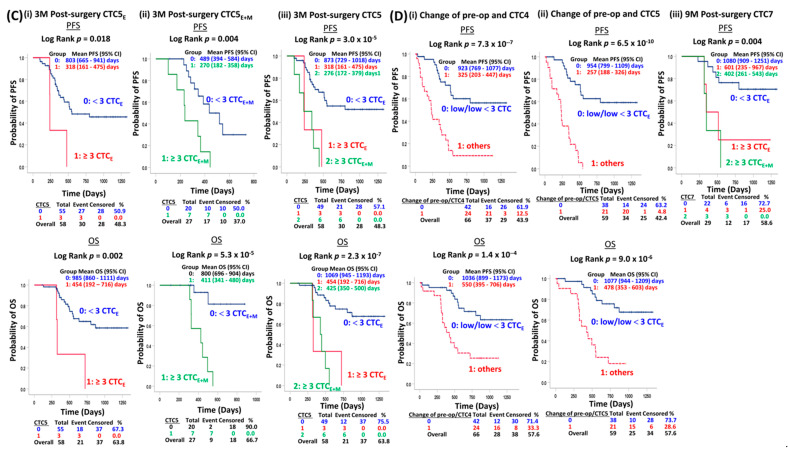
Kaplan–Meier survival analysis of PFS and OS with (**A**) baseline and pre-surgery, (**i**) CTC1_E,_ (**ii**) CTC1_E+M_, (**iii**) CTC1 stratification into three groups 0/1/2: low/high epithelial CTC level/high total CTC level; (**B**) 1M post-surgery (**i**) epithelial CTC4_E_ and (**ii**) total CTC4_E+M_, (**iii**) CTC4 stratification into the following three groups 0/1/2: low/high epithelial CTC level/high total CTC level; (**C**) 3M post-surgery (**i**) epithelial CTC5_E_ and (**ii**) total CTC5_E+M_, (**iii**) CTC5 stratification into three groups 0/1/2: low/high epithelial CTC level/high total CTC level; (**D**) (**i**) the change of CTC status of pre-surgery and CTC4, (**ii**) the change of CTC status of pre-surgery and CTC5, (**iii**) 9M post-surgery: CTC7 stratification into three groups 0/1/2: low/high epithelial CTC level/high total CTC level.

To date, longitudinal monitoring of CTCs in ESCC is lacking, although its prognostic value has been consistently reported [6,8,9,17,20,21,23]. To study the clinical utility of serial monitoring of CTC enumeration for the prognosis of PFS and OS, ESCC patients were dichotomized into favorable and unfavorable groups indicated by the KM survival analysis, as shown in Figure 3 and multivariant COX regression analysis (Table 2).

#### 3.5.1. CTC Analysis at Baseline

At baseline, patients with a high epithelial CTC level (≥2 CTC1E) showed a marginal trend of shorter PFS (*p* = 0.055) but no significant difference for OS (*p* = 0.115) compared to those with a low epithelial CTCE level (<2 CTC1E) (Figure 3A(i)). When we performed CTC enumeration including vimentin, patients with a high total CTC_E+M_ level (≥2 CTC1_E+M_) compared with those with a low total CTC level (<2 CTC1_E+M_) at baseline had a significantly shorter PFS (*p* = 0.006) and OS (*p* = 0.016) (Figure 3A(ii)). Patients were further classified into three groups for risk stratification based on low epithelial CTC level (<2 CTC1_E_) and high epithelial CTC level (≥2 CTC1_E_) into low-risk group 0 and high-risk group 1, respectively. Six patients with a high total CTC1_E+M_ level (≥2 CTC1_E+M_) were classified into the high-risk group 2. Patients in the high-risk groups 1 and 2 at baseline both had a significantly shorter PFS (*p* = 0.001) and OS (*p* = 0.001) (Figure 3A(iii)). The prognostic role of baseline CTC isolated by FAST strategy for patients receiving curative treatment was concordant with earlier studies detecting CTCs with molecular assays examining mRNA levels by RT-PCR, immunomagnetic selection, and ISET strategies [15,17,18,19,20,23,30,31,32,33].

#### 3.5.2. Serial CTC Analysis at Post-Surgery Follow-Up

Patients with a high CTC level (≥3 epithelial CTC4_E_) at 1-month post-surgery showed a marginal trend of shorter PFS (*p* = 0.058), but no significant difference for the OS (*p* = 0.24) compared to those patients with a low CTC level (<3 CTC4_E_) (Figure 3B(i)). Serial epithelial CTC analysis at 3-month post-surgery indicated that patients with a high epithelial CTC level (≥3 CTC5_E_) were significantly associated with shorter PFS (*p* = 0.018) and OS (*p* = 0.002) (Figure 3C(i)), compared to those with a low epithelial CTC level (<3 CTC5_E_). The utilization of vimentin as a mesenchymal marker for CTC enumeration at 1-month and 3-month post-surgery enhanced the prognosis and risk stratification, as more patients carrying aggressive CTCs missed by epithelial markers were identified. At 1 month post-surgery, the ten patients with a high total CTC level (≥3 CTC4_E+M_) had a significantly shorter PFS (*p* = 0.002) and OS (*p* = 0.014) (Figure 3B(ii)). At 3-month post-surgery, the seven patients with high total CTC levels (≥3 CTC5_E+M_) had a significantly shorter PFS (*p* = 0.004) and OS (*p* = 5.3 × 10^−5^) (Figure 3C(ii)). For risk stratification, patients with a low (<3 CTC4_E_/CTC5_E_), high (≥3 CTC4_E_/CTC5_E_) epithelial CTC_E,_ and high total CTC_E+M_ (≥3 CTC4_E+M_/CTC5_E+M_) were categorized into low-risk group 0, and high-risk groups 1 and 2, respectively. The high-risk groups 1 and 2 at 1-month post-surgery had a significantly shorter PFS (*p* = 3.2 × 10^−5^) and a trend of shorter OS (*p* = 0.069) (Figure 3B(iii)). The high-risk groups 1 and 2 at 3-month post-surgery had a significantly shorter PFS (*p* = 3.2 × 10^−5^) and OS (*p* = 2.3 × 10^−7^) (Figure 3C(iii)). The best risk stratification parameters were based on the combined changes of pre-surgery/CTC4 and pre-surgery/CTC5. Patients remained low (<3 CTC_E_) CTC status at both pre-surgery/CTC4 (*p* = 7.3 × 10^−7^; *p* = 1.6 × 10^−4^) (Figure 3D(i)) and pre-surgery/CTC5 (*p* = 6.5 × 10^−10^; *p* = 9 × 10^−6^) (Figure 3D(ii)) with a statistically significant longer PFS and OS compared to other combinations of change. At 9-month post-surgery, when we considered a similar risk stratification strategy of patients into three risk groups, the high-risk groups 1 and 2 at CTC7 had a significantly shorter PFS (*p* = 0.004) compared to risk group 0 (Figure 3D(iii)). The current prospective longitudinal serial CTC monitoring along the course of curative treatment findings first suggests the clinical utility of prognostication and risk stratification of the combined changes of CTC status at pre-surgery/1-month post-surgery and pre-surgery/3-month post-surgery, and post-surgery follow-up timepoints at 1-, 3-, and 9-month status for patients treated by surgery with and without neoadjuvant treatment.

### 3.6. COX Regression Analysis of Independent Prognostic Role of Serial CTC Status

The univariate COX analysis for clinical parameters and CTC levels at multiple CTC timepoints with survival is detailed in Table S7. In the clinical pathological model (CP), the pT staging after resection remained the only independent prognostic factor of relapse and death (Table 2). The multivariate regression model analysis indicated the CTC1, CTC3-CRT, CTC4, CTC5, CTC3/4, and CTC3/5 models with high baseline CTC1 level (HR for CTC1_E_ 6.99, HR for CTC1_E+M_ 19.162, *p* = 2 × 10^−4^), high CTC3_E_ level (HR 3.652, *p* = 0.035), high CTC4_E+M_ level (HR 7.56, *p* = 5.8 × 10^−4^), high CTC5 level (HR for CTC5_E_ 3.641, HR for CTC5_E+M_ 9.946, *p* = 9 × 10^−6^), respectively, independently associated with a higher risk of PFS (Table 2). The COX models CTC1 and CTC5 indicated that a high total baseline CTC1 level (HR for CTC1_E_ 3.665, HR for CTC1_E+M_ 8.719, *p* = 5 × 10^−4^) and high CTC5 level (HR for CTC5_E_ 5.821, HR for CTC5_E+M_ 9.366, *p* = 4 × 10^−5^) independently associated with a higher risk of OS (Table 2).

The model CTC3/4 incorporating the combinations of CTC change between pre-surgery and CTC4 indicated that patients with other unfavorable changes, compared with those remaining with favorable CTC status at both timepoints, were independently associated with a higher risk of relapse and death (PFS HR 3.638, *p* = 8.3 × 10^−4^, OS HR 3.019, *p* = 0.008) (Table 2). Similarly, when we consider the combinations of CTC change between pre-surgery and 3 months post-surgery, the patients with other unfavorable changes had a 6.7-fold higher risk of relapse (*p* = 6.7 × 10^−6^) and 3.9-fold higher risk of death (*p* = 0.002), compared to patients with favorable CTC levels at both timepoints. Among the seven COX models incorporating various timepoints and CTC clusters into the clinical pathological model, the models CTC5 (PFS concordance 0.797, OS concordance = 0.802) and CTC3/5 (PFS concordance 0.803, OS concordance = 0.783) had the best concordance.

Other studies revealed the prognostic role of CTCs in ESCC, only examining either the pre- and post-treatment CTC [8,9,17,18,20,50]. To the best of our knowledge, the current prospective study is the largest longitudinal real-time monitoring of the usefulness of CTC enumeration and treatment outcome of patients receiving curative surgical treatment [31,32]. Our data not only confirmed high CTC levels at pre- and post-treatment but also demonstrated the strong independent prognostic value of longitudinal monitoring of CTC enumeration at baseline, pre-surgery, and post-surgery at 1/3/9 months. Our data also revealed the stratification of patients into two groups based on the change of both pre-surgery/CTC5 status had the highest HR for both PFS and OS and poorer prognoses. The benefits of pre-surgery CTRT treatment for ESCC treatment remain controversial. The baseline high epithelial and total CTC1 levels and epithelial CTC2_E_ levels predicted poor neo-adjuvant treatment efficacy. The association of high epithelial CTC3_E_ level at the end of neoadjuvant CTRT with poor treatment response suggests alternative therapeutic regimens of more aggressive CRT treatment before surgery are needed. The presence of a high CTC level shortly after surgery within three months correlates with a poor prognosis, suggesting adjuvant treatment is needed. Our data now demonstrate the potential clinical usefulness of longitudinal real-time monitoring of CTC levels within the first year of treatment at baseline, pre-III, and end of CTRT as early predictive and prognostic biomarkers. Future studies should further examine the early predictive role of neoadjuvant treatment efficacy and change of baseline/pre-III and baseline/end of CTRT CTC status. CTC analysis at post-surgery 1-, 3-, and 9-month follow-up timepoints also provides useful prognostic information for disease relapse and overall survival. Our data also suggest CTC analysis with additional mesenchymal markers can enhance the sensitive identification of patient subgroups with poor prognosis in ESCC. The current longitudinal CTC enumeration studies is a comprehensive study demonstrating the independent prognostic and risk stratification role of CTCs at baseline, end of CTRT at pre-surgery, 1M, 3M, and 9M post-surgery for ESCC patients receiving curative treatment.

### 3.7. Longitudinal Monitoring of CTC Changes in Response to CTRT and Surgery

**Figure 4:**
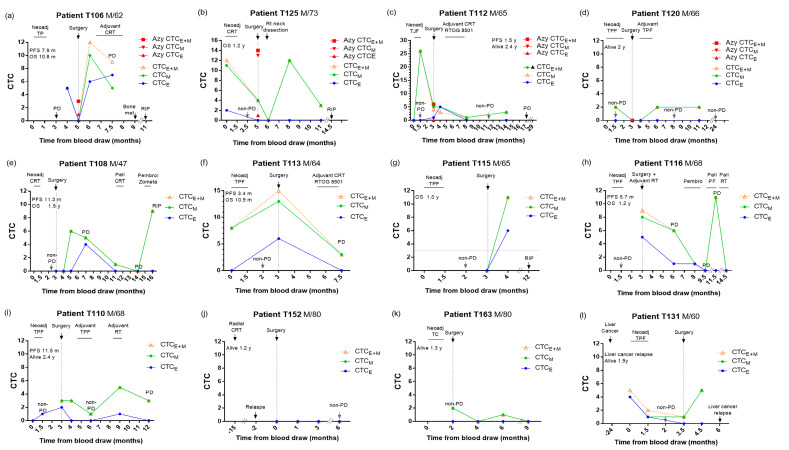
Longitudinal monitoring of epithelial and total CTC changes in response to therapy for 12 patients. M: male; RTOG: Radiation Therapy Oncology Group; TC: docetaxel plus cyclophosphamide; TP: docetaxel plus cisplatin; TPF: docetaxel, cisplatin and fluorouracil.

Imaging and traditional clinical evaluation are insufficient for independent prediction of treatment outcomes. Earlier ESCC CTC studies suggested CTC detected before and after treatment as independent predictors [8,9,10,11,17,51]. Our findings now demonstrate that real-time longitudinal monitoring of epithelial and mesenchymal CTC changes and unfavorable CTC status in multiple timepoints at baseline, pre- and especially, 1-month, 3-month, and 9-month post-operation, are predictive for recurrence and poor prognosis. CTC analysis of twelve patients in surveillance of neoadjuvant CTRT, surgery, and adjuvant CTRT is shown in Figure 4. The AVB CTC levels in four patients are also shown in Figure 4a–d. For instance, patients T106 (Figure 4a), T125 (Figure 4b), and T112 (Figure 4c) had a high level of pre-surgery CTC, AVB CTC, and post-surgery CTC; they died early or with early relapse with poor prognoses. Patient T120 (Figure 4d) with favorable CTC levels at multiple timepoints, including pre-III, pre-surgery (both PB and AVB), 3M, and 8M post-surgery, along with treatment, remained progression-free and alive. For six patients developing drug resistance to treatment and relapse, T106 (Figure 4a), T112 (Figure 4c), T108 (Figure 4e), T113 (Figure 4f), T116 (Figure 4h), and T110 (Figure 4i) the unfavorable total CTC_E+M_ status in PB was concordant to PD detected by imaging. Favorable CTC status in PB was observed for three patients with stable conditions responding to the CRT treatment when the imaging results were non-PD, T120 (Figure 4d), T152 (Figure 4j), and T163 (Figure 4k). However, the CTC counts in PB may not always be consistent with the imaging results. During post-surgery follow-up, when the imaging result was not available, the unfavorable total CTC_E+M_ in PB was elevated 8–10 months in two patients, T125 (Figure 4b) and T115 (Figure 4g), before they died early with OS time of 1.2 and 1 year. For patient T131 with liver cancer two years before ESCC, the unfavorable CTC_E+M_ elevated 1.5 months before the liver cancer relapse, while the ESCC primary tumor was responding (Figure 4l). No CTC was detected at PD of patient T108 at 14 months and T116 at 9.5 months (Figure 4h). This may be partially attributed to the heterogeneity of an aggressive subpopulation of CTCs being missed with the current panel of markers used for enumeration.

We aimed to detect early relapse of patients receiving surgical treatment and chose timepoints at pre- and post-surgery 1-month and 3-month intervals in the first-year follow-up. This real-time monitoring would allow patients to have an earlier switch to the next line of therapy and tailor a personalized treatment plan. Thus, the non-invasive longitudinal monitoring of the change of CTC level from favorable to unfavorable in the PB can dynamically track tumor development and reflect real-time changes for ESCC surveillance. CTC real-time monitoring holds great promise as a potential biomarker for minimal residual disease and assessment of treatment efficacy. However, currently, the use of CTC in clinics is limited by the high heterogeneity of CTC populations, low detection rate, high cost, and great individual variations. Future studies should further identify additional markers hallmarking aggressive cancer stem cell-like CTC subpopulations resulting in metastasis and poor prognoses.

The relatively modest sample size in the current cohort study warrants further larger cohort validation to assess the clinical usefulness of the total CTC status at baseline, pre-operation, and 1M, 3M, and 9M post-operation, as well as the change of CTC status between the pre-operation/1M post-operation, and pre-operation/3M post-operation. A limitation of this study was 28 patients missed the baseline CTCs for analysis, although earlier studies had demonstrated its clinical utility [31,32]. Another limitation was the underestimation of total CTCs due to our size-based capture platform’s biased results towards larger CTCs. CTC underestimation is related to the heterogeneity of aggressive CTC surface markers.

## 4. Conclusions

Longitudinal CTC real-time monitoring suggested the presence of CTC clusters, unfavorable CTC status at baseline, 1-month and 3-month post-surgery, and the change of CTC status between pre-surgery/1-month post-surgery, pre-surgery/3-month post-surgery, and pT staging after resection are independent prognostic factors of poor prognosis for locally advanced ESCC patients receiving surgical treatment with or without CTRT. For the subgroup of patients treated by CTRT before surgery, the unfavorable CTC status at the end of CTRT, sex, and pT staging are independent prognosticators. Moreover, the unfavorable post-CTRT CTC levels may be potential predictive biomarkers for the prediction of neoadjuvant treatment efficacy. More CTCs were released from patients treated by CTRT compared to upfront surgery. Our findings also suggested CTC enumeration in ESCC, including mesenchymal markers, improved the CTC detection rate and enhanced the capture of the aggressive CTC subsets undergoing EMT. Future studies should consider the cost-effectiveness and search for additional aggressive ESCC tumor markers to improve false-negative results due to the unsatisfactory sensitivity and accuracy for ESCC CTC isolation and enumeration. Our study recommends several of the most useful timepoints taken during pre-treatment, post-treatment/pre-surgery, and 1-month and 3-month post-surgery. The CTC dynamic changes along treatment may supplement clinical imaging to track minimal residual diseases. Longitudinal real-time CTC monitoring provides useful prognostic and predictive information as potential minimally invasive blood-based biomarkers for treatment efficacy, disease relapse, and survival for advanced ESCC. In the future, additional large and long-term multicentered studies or clinical trials will help substantiate the clinical efficacy of longitudinal CTC real-time monitoring.

## Figures and Tables

**Table 1:** Baseline characteristics of CTC bloods for serial monitoring of CTC counts in ESCC patients.

Clinical Parameters	Patients (*n* = 86)	CTC1_E_ (*n* = 60)		CTC3_E_ (*n* = 43)		CTC4_E_ (*n* = 63)		CTC5_E_ (*n* = 58)		CTC1_E+M_ (*n* = 59)		CTC3_E+M_ (*n* = 43)		CTC4_E+M_ (*n* = 63)		CTC5_E+M_ (*n* = 58)	
		≥2	<2	≥2	<2	≥3	<3	≥3	<3	≥2	<2	≥2	<2	≥3	<3	≥3	<3
Median age (range)	69 ± (30–87)			^c^*p* = 0.119													
<69	41 (47.7%)	6	19	7	19	4	26	3	24	8	17	9	17	6	24	6	21
≥69	45 (52.3%)	5	30	1	16	5	28	0	31	9	26	5	13	8	25	3	28
Sex																	
Male	64 (74.4%)	10	35	7	25	6	41	2	42	14	31	12	21	9	38	7	37
Female	22 (25.6%)	1	14	1	10	3	13	1	13	3	12	2	9	5	11	2	12
G category ^a^																	
GX	36 (41.9%)	4	22	4	17	3	21	3	20	7	19	5	16	5	19	4	19
G1/G2	38 (44.2%)	5	21	3	15	4	27	0	27	7	19	7	12	6	25	4	23
G3	12 (14.0%)	2	6	1	3	2	6	0	8	3	5	2	2	3	5	1	7
Tumor Location																	
Upper/Middle	57 (66.3%)	8	32	6	23	5	35	2	32	13	27	10	20	7	33	6	28
Lower	29 (33.7%)	3	17	2	12	4	19	1	23	4	16	4	10	7	16	3	21
Stage ^b^																	
Early: I + II	28 (32.6%)	5	18	1	11	2	21	1	22	6	17	3	9	3	20	3	20
Late: III + IV	54 (62.8%)	5	30	7	21	7	29	2	30	10	25	11	18	11	25	6	26
Unknown	4 (4.7%)	-		-		-		-		-		-		-		-	
pT (*n* = 73)				^c^*p* = 0.156													
0–2	33 (45.2%)	7	21	1	18	2	29	0	21	7	14	4	15	4	27	2	19
3–4	40 (54.8%)	4	17	4	12	6	25	3	32	9	19	7	10	9	22	7	28
pN (*n* = 73)				^c^*p* = 0.069													
0	39 (53.4%)	5	23	1	19	5	27	2	25	11	17	6	14	7	25	4	23
1–3	34 (46.6%)	1	20	5	11	3	26	1	27	6	15	6	11	6	23	5	23
Treatment														^c^*p* = 0.126		**^c^*p* = 0.008**	
CRT ± surgery	54 (62.8%)	7	22	8	35	6	31	3	32	8	23	14	30	11	26	9	26
Upfront surgery	32 (37.2%)	4	27	NA	NA	3	23	0	23	9	20	NA	NA	3	23	0	23
Distant Metastasis																	
No	77 (89.5%)	11	44	7	32	9	49	2	50	9	44	13	27	14	44	7	45
Yes	9 (10.5%)	0	5	1	3	0	5	1	5	0	5	1	3	0	5	2	4

^a^ Squamous cell carcinoma G category: GX, G1, G2, G3 = Differentiation cannot be assessed, well, moderately, and poorly differentiated, respectively. Only one patient was G1 and, therefore, G1 was combined with G2. ^b^ 8th edition of American Joint Committee on Cancer (AJCC) TNM system for Cancer Staging. CTC1_E_: baseline epithelial CTC, CTC3_E_: post-treatment/pre-surgery epithelial CTC, CTC4_E_: 1-month post-surgery epithelial CTC, CTC5_E_: 3-month post-surgery epithelial CTC. CTC1_E+M_: baseline total CTC, CTC3_E+M_: post-treatment/pre-surgery total CTC, CTC4_E+M_: 1-month post-surgery total CTC, CTC5_E+M_: 3-month post-surgery total CTC. CTC2_E_, CTC6_E_–CTC12_E_ and CTC6_E+M_–CTC12_E+M_, are not included as *n* < 20 or only <3 patients with high CTC status. ^c^ = Fisher exact test, 2-sided. Bold if *p* < 0.05.

**Table 2:** Multivariate COX regression analysis of clinical pathological (CP) parameters and CTC counts at baseline, pre- and post-operation with progression-free and overall survival.

Models	Variables	PFS			OS		
		HR (95% CI)	*p*-Value	Concordance	HR (95% CI)	*p*-Value	Concordance
CP ^a^	pT (3 + 4 vs. 1 + 2 ref) (*n* = 73)	4.097 (1.79–9.36)	**8.0 × 10^−4^**	0.696	3.190 (1.28–7.98)	**0.013**	0.655
CTC1 ^b^	pT (3 + 4 vs. 1 + 2 ref) (*n* = 49) Sex	3.319 (1.23–9.0) 8.408 (1.65–42.94)	**0.018** **0.011**	0.782	- -	- -	0.76
	Baseline CTC1 level (≥2 CTC_E_ vs. <2 CTC_E_ ref) (≥2 CTC_E+M_ vs. <2 CTC_E_ ref)	6.99 (2.10–23.22) 19.162 (4.72–77.74)	**2.0 × 10^−4^** **0.0015** **3.6 × 10^−5^**		3.665 (1.34–10.04) 8.719 (2.69–28.25)	**5.0 × 10^−4^** **0.012** **3.1 × 10^−4^**	
CTC3-CRT ^c^	pT (3 + 4 vs. 1 + 2 ref) (*n* = 35) Sex:strata(tgroup1)	5.90 (1.48–23.51) 0.03 (0.002–0.45)	**0.012** **0.012**	0.793	4.648 (0.90–23.99) 0.130 (0.01–1.30)	0.067 0.082	0.737
	CTC3_E_ count at the end of CTRT (≥2 vs. <2 CTCs ref) (≥3 vs. <3 CTCs ref)	3.652 (1.1–12.14) N/A	**0.035** N/A		N/A 3.50 (0.67–18.35)	N/A 0.139	
CTC4 ^d^	pT (3 + 4 vs. 1 + 2 ref) (*n* = 62)	3.222 (1.23–8.46)	**0.017**	0.776	-	-	0.676
	Sex:strata(tgroup1)	0.07 (0.006–0.70)	**0.024**		-	-	
	CTC4 count at post-surgery 1M (≥3 CTC_E_ vs. <3 CTC_E_ ref) (≥3 CTC_E+M_ vs. <3 CTC_E_ ref)	1.822 (0.67–4.98) 7.56 (3.39–23.91)	**4.0 × 10**^**−4**^ 0.242 **5.8 × 10^−4^**		1.536 (0.48–4.88 2.902 (0.79–10.62)	0.1 0.467 0.107	
CTC5 ^e^	pT (3 + 4 vs. 1 + 2 ref) (*n* = 56)	6.792 (2.04–22.58)	**0.002**	0.797	-	-	0.802
	CTC5 count at post-surgery 3M (≥3 CTC_E_ vs. <3 CTC_E_ ref) (≥3 CTC_E+M_ vs. <3 CTC_E_ ref)	3.641 (1.00–13.25) 9.946 (3.49–28.34)	**9.0 × 10^−6^** **0.0499** **1.7 × 10^−5^**		5.821 (1.52–22.32) 9.366 (3.11–28.18)	**4.0 × 10^−5^** **0.01** **6.9 × 10^−5^**	
CTC3/4 ^f^	pT (3 + 4 vs. 1 + 2 ref) (*n* = 62)	3.086 (1.16–8.22)	**0.024**	0.783	-	-	0.739
	Change of pre-surgery/CTC4 Others vs. favorable change (<3 CTC_E_) pre-surgery/CTC4 ref)	3.638 (1.71–7.75)	**8.3 × 10^−4^**		3.019 (1.34–6.82)	**0.008**	
CTC3/5 ^g^	pT (3 + 4 vs. 1 + 2 ref) (*n* = 56)	4.259 (1.47–12.32)	**0.007**	0.803	-	-	0.783
	Change of pre-surgery/CTC5 Others vs. favorable change (<3 CTC_E_) pre-surgery/CTC5 ref)	6.662 (2.92–15.21)	**6.7 × 10^−6^**		3.913 (1.66–9.21)	**0.002**	
CTC3CL ^h^	pT (3 + 4 vs. 1 + 2 ref) (*n* = 63)	3.639 (1.48–8.93)	**0.005**	0.749	3.022 (1.14–8.03)	**0.027**	0.662
					-	-	
	CTC clusters (Yes vs. No ref)	2.539 (0.94–6.89)	0.068				

^a^ CP COX model for PFS includes a stage at diagnosis and pT as covariates and sex as a time-varying covariate; the CP COX model for OS includes pT and sex as covariates. ^b^ CTC1 COX model adding baseline CTC_E+M_ count to the CP COX model. ^c^ CTC3-CRT COX model adding CTC3_E_ at pre-surgery to the CP COX model. ^d^ CTC4 COX model adding CTC4_E+M_ to the CP COX model. ^e^ CTC5 COX model adding CTC5_E+M_ to the CP COX model. ^f^ CTC3/4 COX model adding the change of pre-surgery/CTC4 status to the CP COX model. ^g^ CTC3/5 COX model adding the change of pre-surgery/CTC5 status to the CP COX model. ^h^ CTC3CL COX model adding pre-surgery CTC clusters to the CP COX model. Bold if *p*-value < 0.05. N/A: Not applicable.

## Data Availability

The data presented in this study are available on request from the corresponding author.

## Supplementary Materials

The following supporting information can be downloaded at: https://www.mdpi.com/article/10.3390/cancers15030832/s1, Figure S1: Flow chart of patient selection; Figure S2: Timeline for serial blood specimen collection; Figure S3: Kaplan–Meier analysis of treatment with PFS and OS; Figure S4: Higher total CTC_E+M_ counts at baseline, 1M, 3M, 6M, and 9M post-operation, statistically significant differences were observed at baseline and 3M post-operation; Figure S5: Summary of serial analysis of positive CTC detection rate; Table S1: Blood sampling at different timepoints; Table S2: ROC cut off of CTC and survival; Table S3: Association of pre-III CTC2_E_ with poor pre-surgery treatment response in subgroup of nine patients with adjuvant treatment after surgery; Table S4: The range, median, and positive detection rates of epithelial CTC_E_ at CTC1 to CTC13; Table S5: The range, median, and positive detection rates of total CTC_E+M_ at CTC1 to CTC13; Table S6: Patients with recurrent CTC clusters; Table S7: Univariate COX regression analysis of clinical pathological parameters and CTC counts at baseline, pre-, and post-operation with progression-free and overall survival.
