# Supplementary material for: Circulating Tumor Cell Enumeration for Serial Monitoring of Treatment Outcomes for Locally Advanced Esophageal Squamous Cell Carcinoma
Source: Cancers (Basel). 2023 Jan 29;15(3):832. doi: 10.3390/cancers15030832 (PMC9913330; doi:10.3390/cancers15030832)
Supplement: Supplementary file 1 [file cancers-15-00832-s001.zip › cancers-2172778-supplementary.pdf]

List of Supplementary Figures and Tables

Supplementary Figure S1: Flow chart of patient selection

Supplementary Figure S2: Timeline for serial blood specimen collection

Supplementary Figure S3: Kaplan-Meier analysis of treatment with PFS and OS

Supplementary Figure S4: Higher total CTC_{E+M} counts at baseline, 1M, 3M, 6M and 9M post-operation, statistically significant differences were observed at baseline and 3M post-operation

Supplementary Figure S5: Summary of serial analysis of positive CTC detection rate

Supplementary Table S1: Blood sampling at different timepoints

Supplementary Table S2: ROC cut off of CTC and survival

Supplementary Table S3: Association of pre-III $CTC2_E$ with poor pre-surgery treatment response in subgroup of 9 patients with adjuvant treatment after surgery

Supplementary Table S4: The range, median and positive detection rates of epithelial CTC_E at CTC1 to CTC13

Supplementary Table S5: The range, median and positive detection rates of total CTC_{E+M} at CTC1 to CTC13

Supplementary Table S6: Patients with recurrent CTC clusters

Supplementary Table S7: Univariate COX regression analysis of clinical pathological parameters and CTC counts at baseline, pre- and post-operation with progression-free and overall survival.

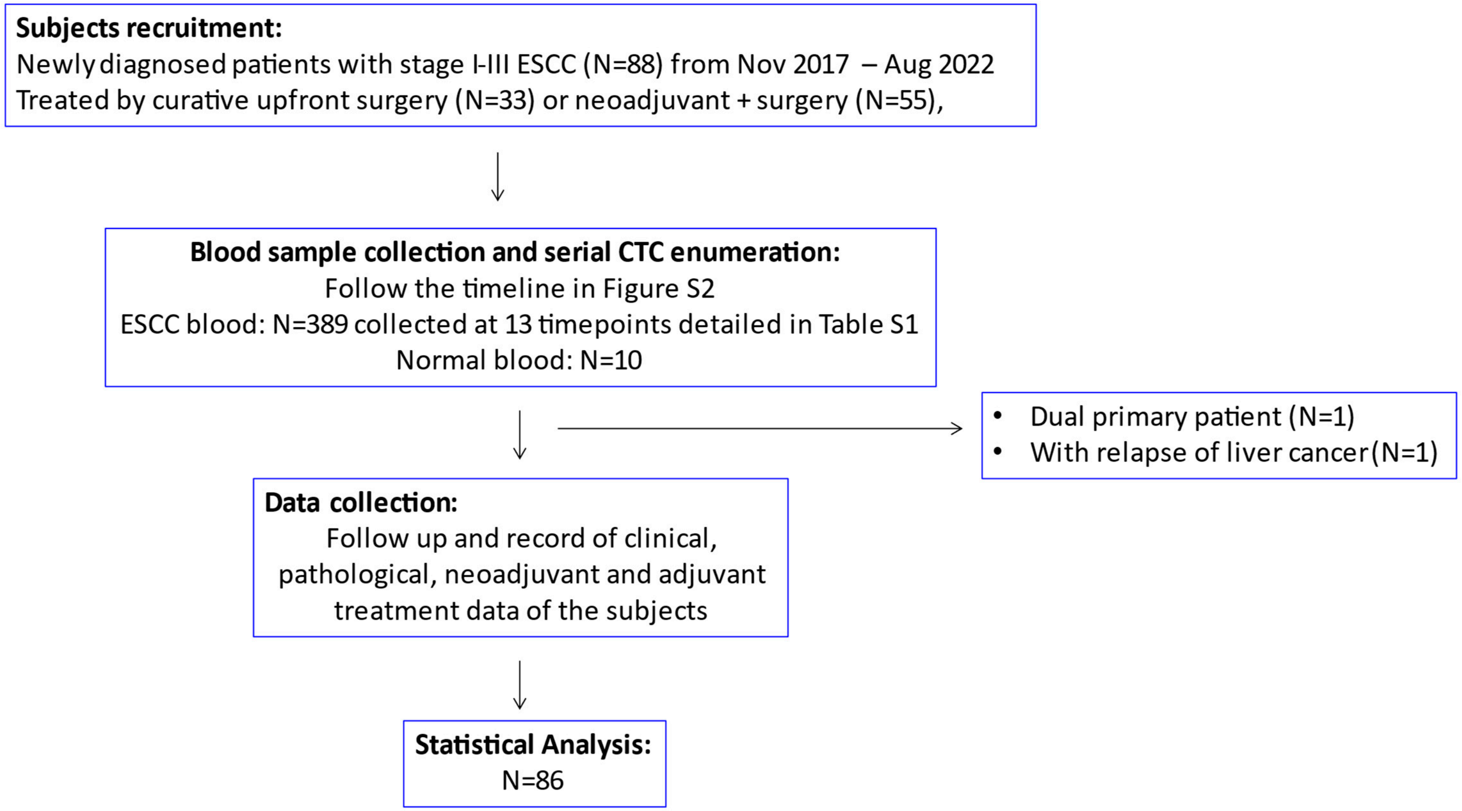

One patient was recruited for two lines of treatment after progression. CT treatment included paclitaxel and carboplatin (TC) or platinum-based (cisplatin and 5-fluorouracil (PF) or docetaxel (TPF)) chemotherapy.

Figure S1: Flow chart of patient selection

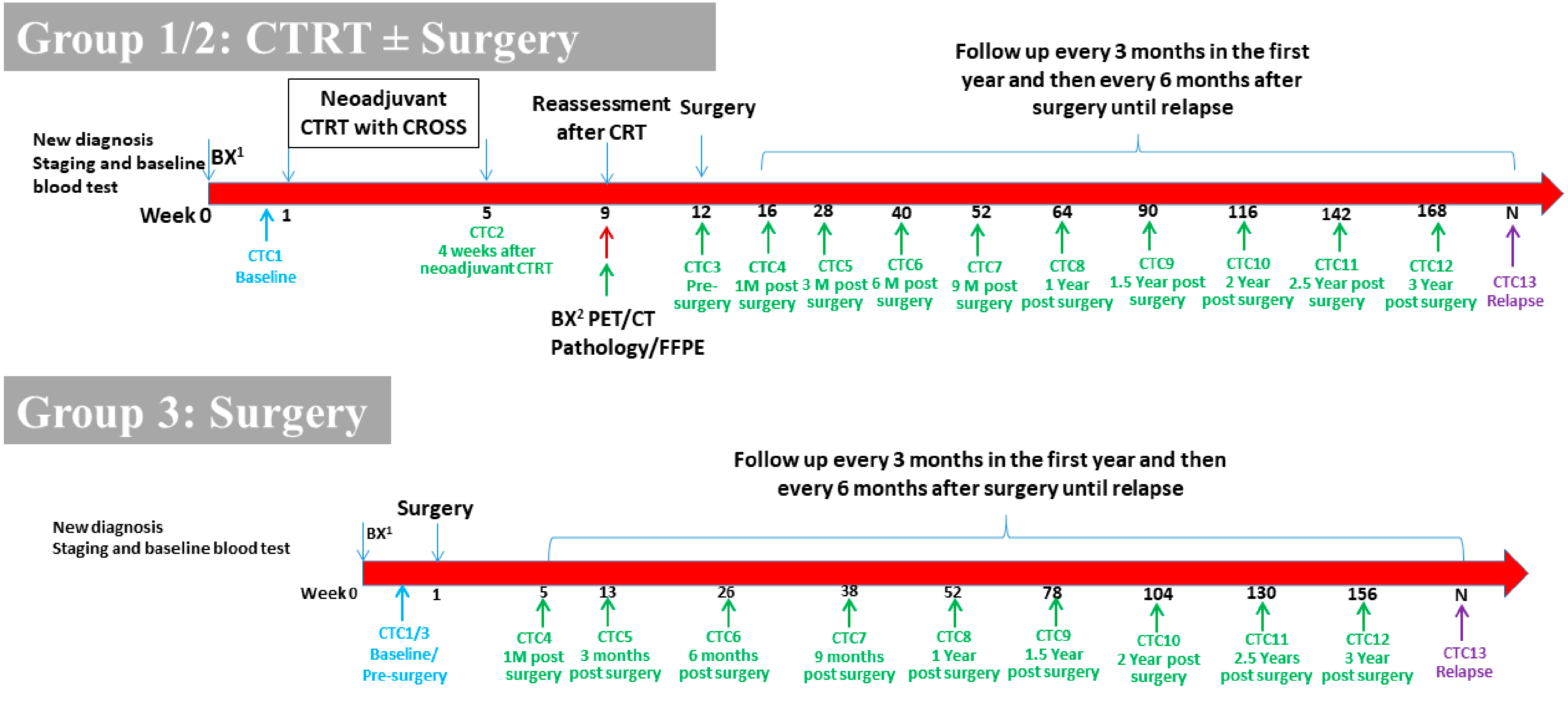

Remarks:
CTC2: Reassessment at about 4 weeks after neoadjuvant CRT
CTC3: ~4 Weeks after surgery as early predictive biomarkers
CTC4-7: ~3/6/9/12 months post surgery for some cases, CTC8-12: ~1/1.5/2/2.5/3 years post surgery for some cases, CTC4-12 = CTCN, whichever comes earlier
CTCN: Relapse
BX1: For patients with more than three bites, one bite was collected. For patients without biopsy, the FFPE blocks were collected from pathology department if available.

1

Supplementary Figure S2: Timeline for serial blood specimen collection

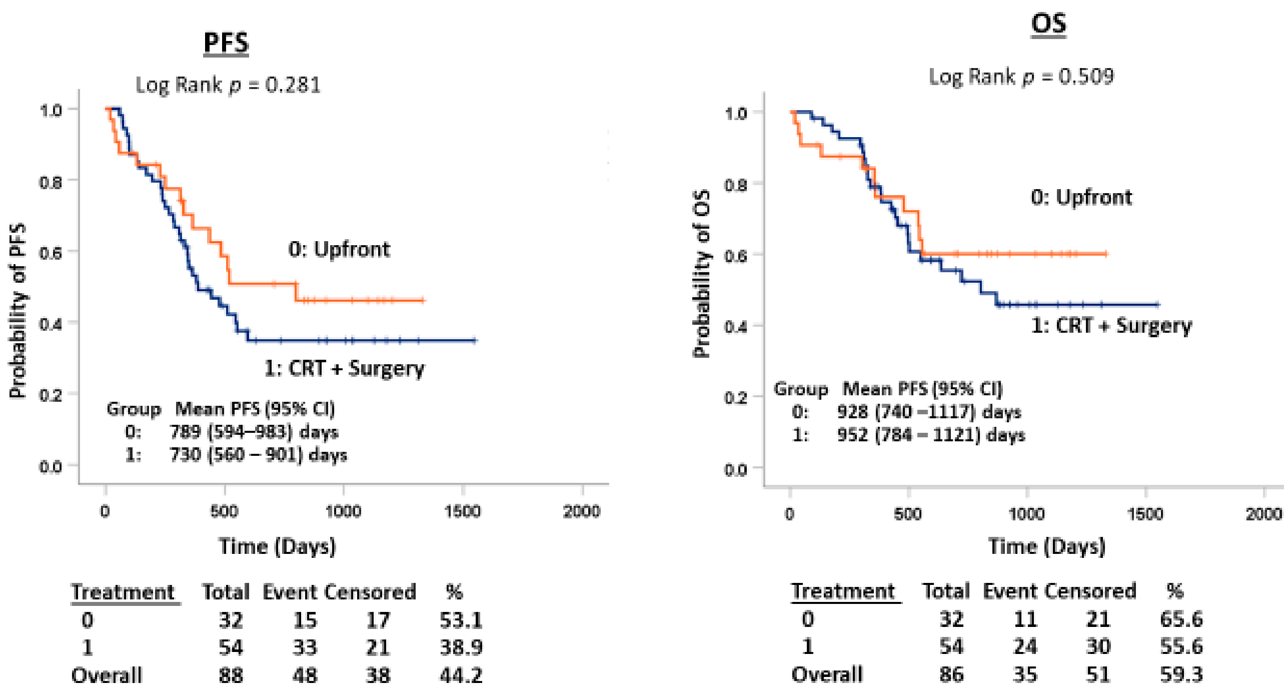

Supplementary Figure S3: Kaplan Meier analysis of treatment with PFS and OS

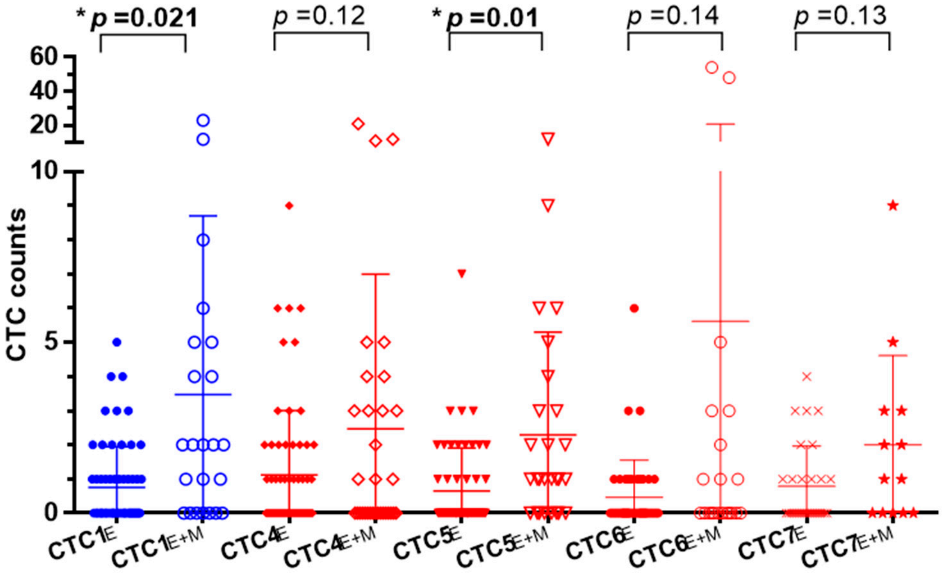

Supplementary Figure S4: Higher total CTC_{E+M} counts at baseline, 1M, 3M, 6M and 9M post-operation, statistically significant differences were observed at baseline and 3M post-operation.

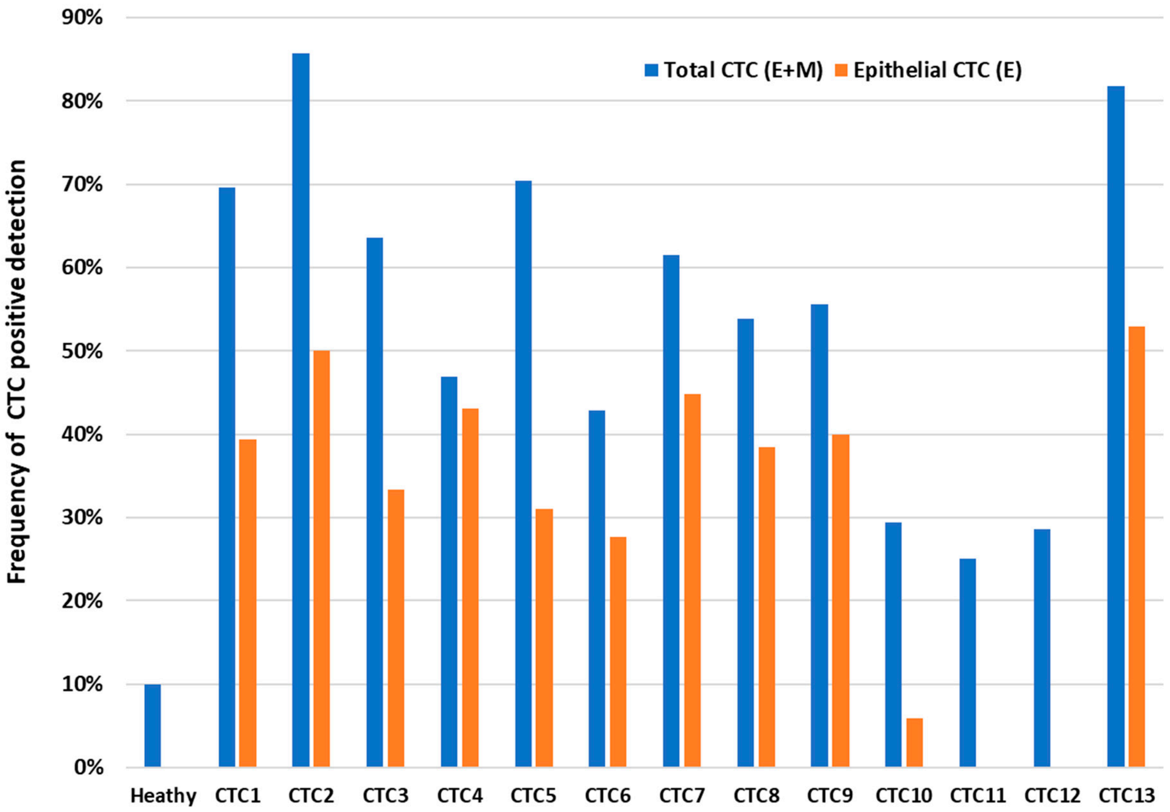

Supplementary Figure S5: Summary of serial analysis of positive CTC detection rate

Supplementary Table S1: Blood sampling at different timepoints

Timepoints	CRT + surgery (N = 55)	Surgery (N = 33)	Total (N = 88)
CTC1*	32	29	61
CTC2	10	NA	10
CTC3	45	NA	45
CTC4	39	26	65
CTC5	35	23	58
CTC6	27	20	47
CTC7	14	15	29
CTC8	13	13	26
CTC9	6	4	10
CTC10	10	7	17
CTC11	6	6	12
CTC12	3	4	7
CTC13	11	6	17
Total[a]	241	148	389

*27 patients missed the baseline. The baseline CTC were missing in 20 patients received neoadjuvant CRT treatment at other hospitals and then transfer to QMH for the surgical resection. Seven patients did not have enough blood for CTC enumeration.

[a]15 patients at relapsed timepoint overlapped with CTC4-9.

Supplementary Table S2: ROC analysis of CTC and survival

	PFS				OS			
CTC timepoints	AUROC	Threshold	Sensitivity	Specificity	AUROC	Threshold	Sensitivity	Specificity
$CTC1_{E+M}$	0.734	1.5	0.688	0.833	0.734	1.5	0.688	0.833
$CTC3_{E}$	0.69	1.5	0.308	1	0.57	2.5	0.2	0.913
$CTC4_{E+M}$	0.768	2.5	0.526	1	0.708	2.5	0.636	0.842
$CTC5_{E+M}$	0.8	2.5	0.438	1	0.876	2.5	0.778	1
$CTC7_{E+M}$	0.638	2.5	0.5	1	NA			

Supplementary Table S3: Association of pre-III $CTC2_E$ with poor pre-surgery treatment response in subgroup of 9 patients with adjuvant treatment after surgery

End of CTRT Imaging Reassessment	Positive $CTC2_E$	Negative $CTC2_E$	Fisher Exact Test, 2-sided *p*
Good response<br>- 0 CR<br>- 6 PR	1	5	**0.048**
Poor response<br>- 1 SD<br>- 2 PD	3	0	
	4	5	

Supplementary Table S4: The range, median and positive detection rates of epithelial CTC_E at CTC1 to CTC13

Statistics	CTC1 Baseline$_E$	CTC2 Pre-III$_E$	CTC3 Pre-surgery$_E$	CTC4 1M post-surgery$_E$	CTC5 3M post-surgery$_E$	CTC6 6M post-surgery$_E$	CTC7 9M post-surgery$_E$	CTC8 12M post-surgery$_E$	CTC9 18M post-surgery$_E$	CTC10 24M post-surgery$_E$	CTC11 30M post-surgery$_E$	CTC12 36M post-surgery$_E$	CTC13 Relapse$_E$
Number of values	61	10	45	65	58	47	29	26	10	17	12	7	17
Minimum	0.0	0.0	0.0	0.0	0.0	0.0	0.0	0.0	0.0	0.0	0.0	0.0	0.0
Median	0.0	0.5	0.0	0.0	0.0	0.0	0.0	0.0	0.0	0.0	0.0	0.0	1.0
Maximum	5.0	42.0	9.0	9.0	7.0	6.0	4.0	3.0	2.0	1.0	0.0	0.0	8.0
Mean	0.8	5.0	1.0	1.1	0.7	0.5	0.8	0.5	0.5	0.1	0.0	0.0	2.1
Std. Deviation	1.2	13.1	2.1	1.9	1.3	1.1	1.2	0.8	0.7	0.2	0.0	0.0	2.6
number of samples with detectable CTC_E	24	5	15	28	18	13	13	10	4	1	0	0	9
Positive CTC_E detection rate	39.3%	50.0%	33.3%	43.1%	31.0%	27.7%	44.8%	38.5%	40.0%	5.9%	0.0%	0.0%	52.9%

Supplementary Table S5: The range, median and positive detection rates of total CTC_{E+M} at CTC1 to CTC13

Statistics	CTC1 Baseline $_{E+M}$	CTC2 Pre-III$_{E+M}$	CTC3 Pre-op$_{E+M}$	CTC4 1M post-surgery$_{E+M}$	CTC5 3M post-surgery$_{E+M}$	CTC6 6M post-surgery$_{E+M}$	CTC7 9M post-surgery$_{E+M}$	CTC8 12M post-surgery$_{E+M}$	CTC9 18M post-surgery$_{E+M}$	CTC10 24M post-surgery$_{E+M}$	CTC11 30M post-surgery$_{E+M}$	CTC12 36M post-surgery$_{E+M}$	CTC13 Relapse$_{E+M}$
Number of values	23	7	22	32	27	21	13	13	9	17	12	7	11
Minimum	0.0	0.0	0.0	0.0	0.0	0.0	0.0	0.0	0.0	0.0	0.0	0.0	0.0
Median	2.0	2.0	1.5	0.0	1.0	0.0	1.0	1.0	1.0	0.0	0.0	0.0	3.0
Maximum	23.0	99.0	284.0	21.0	12.0	54.0	9.0	4.0	1.0	10.0	1.0	2.0	284.0
Mean	3.5	19.4	15.2	2.5	2.3	5.6	2.0	1.0	0.6	0.9	0.3	0.6	28.9
Std. Deviation	5.2	36.2	60.1	4.5	3.0	15.2	2.6	1.2	0.5	2.4	0.5	1.0	84.6
number of samples with detectable CTC_{E+M}	16	6	14	15	19	9	8	7	5	5	3	2	9
Positive CTC_{E+M} detection rate	69.6%	85.7%	63.6%	46.9%	70.4%	42.9%	61.5%	53.8%	55.6%	29.4%	25.0%	28.6%	81.8%

Supplementary Table S6: Patients with recurrent CTC clusters

Patient ID	Timepoints with CTC clusters
T112	CTC2 (PB) and CTC3 (both PB and Azygos blood)
T113	CTC1 (PB) and CTC3 (PB)
T125	CTC1 (PB) and CTC3 (both PB and Azygos blood)
T193	CTC2 (PB) and CTC3 (PB)

Supplementary Table S7: Univariate COX regression analysis of clinical pathological parameters and CTC counts at baseline, pre- and post-surgery with progression-free and overall survival.

Variables	PFS HR (95% CI)	*p*-Value	OS HR (95% CI)	*p*-Value
Age	1.010 (0.98 – 1.04)	0.539	0.997 (0.96 – 1.03)	0.856
Gender (M vs F ref)	**2.890 (1.22 – 6.87)**	**0.016**	**5.005 (1.53 – 16.42)**	**0.008**
L cat (Others vs Lower ref)	1.384 (0.72 – 2.65)	0.352	1.408 (0.68 – 2.94)	0.361
G cat (G3 vs G1+G2 ref)	1.231 (0.49 – 3.09)	0.657	0.877 (0.29 – 2.61)	0..813
Stage at diagnosis (III + IV vs I+II ref)	**2.66 (1.30 – 5.44)**	**0.007**	2.13 (0.99 – 4.60)	0.055
pT (3+4 vs 1+2 ref)	**4.108 (1.86 – 9.09)**	**4.9×10^{-4}**	**4.367 (1.76 – 10.86)**	**0.002**
pN (1-3 vs 0 ref)	1.711 (0.87 – 3.38)	0.122	1.545 (0.72 – 3.31)	0.263
Distant metastasis (Yes vs No ref)	1.945 (0.86 – 4.38)	0.108	2.310 (0.95 – 5.59)	0.064
Baseline CTC1 level (n = 58)		**0.002**		**0.004**
High epithelial CTC level (≥3 CTC_E vs 0-2 CTC_E ref)	**4.846 (1.80 – 13.08)**	**0.002**	**4.389 (1.62 – 11.93)**	**0.004**
High total CTC level (≥3 CTC_{E+M} vs 0-2 CTC_E ref)	**3.708 (1.08 – 12.69)**	**0.037**	**4.059 (1.16 – 14.24)**	**0.029**
Pre-surgery CTC_E count (All patients)				
(≥2 CTC_E vs 0-1 CTC_E ref) (n = 72)	**3.360 (1.72 – 6.57)**	**3.9×10^{-4}**	1.982 (0.91 – 4.31)	0.084
(≥3 CTC_E vs 0-2 CTC_E ref) (n = 72)	**9.600 (4.35 – 21.17)**	**2.1×10^{-8}**	**4.956 (2.18 – 11.28)**	**1.4×10^{-4}**
$CTC3_E$ count at end of CTRT (Neo-adjuvant treatment)				
(≥2 vs 0-1 CTCs ref) (n = 43)	**6.271 (2.60 – 15.11)**	**4.3×10^{-5}**	1.954 (0.71 – 5.40)	0.196
(≥3 vs 0-2 CTCs ref) (n = 43)	**43.183 (8.35 – 223.37)**	**7.0×10^{-6}**	**3.485 (1.15 – 10.55)**	**0.027**
CTC4 count at post-surgery 1M (n = 60)		**0.001**		**0.020**
(≥3 CTC_E vs 0-2 CTC_E ref)	**2.775 (1.15 – 6.70)**	**0.023**	2.099 (0.75 – 5.90)	0.159
(≥3 CTC_{E+M} vs 0-2 CTC_E ref)	**8.602 (2.33 – 31.78)**	**0.001**	**8.480 (1.67 – 42.96)**	**0.010**
CTC5 count at post-surgery 3M (n = 57)		**2.0×10^{-4}**		**3.0×10^{-4}**
(≥3 CTC_E vs 0-2 CTC_E ref)	**5.478 (1.55 – 19.41)**	**0.008**	**7.227 (2.00 – 26.11)**	**0.003**
(≥3 CTC_{E+M} vs 0-2 CTC_E ref)	**6.503 (2.42 – 17.46)**	**2.0×10^{-4}**	**6.840 (2.24 – 20.88)**	**7.3×10^{-4}**
Change of pre-surgery and CTC4 (n=64)				
Others vs favorable status (0-2 CTC_E) at both pre-surgery and CTC4 ref)	**4.371 (2.21 – 8.65)**	**2.3×10^{-5}**	**4.167 (1.94 – 8.96)**	**2.6×10^{-4}**
Change of pre-surgery and CTC5 (n=56)				
Others vs favorable status (0-2 CTC_E) at both pre-surgery and CTC5 ref)	**9.366 (4.15 – 21.13)**	**7.1×10^{-8}**	**8.136 (3.49 – 18.99)**	**1.3×10^{-6}**
Pre-surgery CTC clusters (Yes vs No ref) (n=85)	**2.974 (1.17 – 7.59)**	**0.023**	2.263 (0.68 – 7.49)	0.181

Univariate COX regression analysis

Pearson chi square and Fisher's exact tests were used for comparison between CTC status and categorical clinicopathological factors. Student t test was used for comparison of CTC numbers. Among the clinical pathological parameters analysed by the univariate COX regression, gender, clinical tumor staging at diagnosis, and the pathological primary tumour (pT) staging after resection were significantly associated with PFS and OS (p<0.05), while no significant association was observed between age, location of primary tumour (L cat), differentiation status (G cat), pathological regional lymph node (pN) staging after resection and distant metastasis and PFS and OS.

The presence of pre-surgery clusters, CTC level at baseline, pre-operation, and end of CRT for patients receiving neoadjuvant treatment, CTC4, CTC5, and change of pre-surgery and CTC5 CTC levels were associated with PFS and OS by univariate COX analysis (p<0.05).
